# Disentangling root system responses to neighbours: identification of novel root behavioural strategies

**DOI:** 10.1093/aobpla/plv059

**Published:** 2015-05-27

**Authors:** Pamela R. Belter, James F. Cahill

**Affiliations:** Department of Biological Sciences, University of Alberta, Edmonton, AB, Canada T6G 2E9

**Keywords:** Coexistence, competition, habitat use and separation, plant behaviour, plant foraging, plant strategies, root ecology

## Abstract

Plants live in a social environment, with interactions among neighbours a ubiquitous aspect of life. Though many of these interactions occur in the soil, our understanding of how plants alter root growth and the patterns of soil occupancy in response to neighbours is limited. This is in contrast to a rich literature on the animal behavioural responses to changes in the social environment. For plants, root behavioural changes that alter soil occupancy patterns can influence neighbourhood size and the frequency or intensity of competition for soil resources; issues of fundamental importance to understanding coexistence and community assembly. Here we report a large comparative study in which individuals of 20 species were grown with and without each of two neighbour species. Through repeated root visualization and analyses, we quantified many putative root behaviours, including the extent to which each species altered aspects of root system growth (e.g. rooting breadth, root length, etc.) in response to neighbours. Across all species, there was no consistent behavioural response to neighbours (i.e. no general tendencies towards root over-proliferation nor avoidance). However, there was a substantial interspecific variation showing a continuum of behavioural variation among the 20 species. Multivariate analyses revealed two novel and predominant root behavioural strategies: (i) size-sensitivity, in which focal plants reduced their overall root system size in response to the presence of neighbours, and (ii) location-sensitivity, where focal plants adjusted the horizontal and vertical placement of their roots in response to neighbours. Of these, size-sensitivity represents the commonly assumed response to competitive encounters—reduced growth. However, location sensitivity is not accounted for in classic models and concepts of plant competition, though it is supported from recent work in plant behavioural ecology. We suggest that these different strategies could have important implications for the ability of a plant to persist in the face of strong competitors, and that location sensitivity may be a critical behavioural strategy promoting competitive tolerance and coexistence.

## Abstract (precis)

Plants live in a social environment, interacting with the roots and shoots of neighbours. Life with neighbours is a chronic stress, with different behaviours altering the social dynamics. To understand root behavioural strategies in response to neighbours, we observed root growth for 20 species. There was no single response, and instead a continuum of responses from avoidance to aggregation near neighbours. Species were capable of two strategies, (1) location-sensitivity, adjusting the vertical and horizontal placement of roots, and (2) size-sensitivity, reducing root system size. Overall, there is surprising complexity in how plants respond to social environments, with implications for resource use, coexistence, and production.

## Introduction

The close proximity of neighbours, combined with strongly overlapping resource requirements, results in competition for limiting resources being a commonly experienced ecological interaction among plants. Competition can greatly reduce individual fitness and alter evolutionary trajectories (Keddy 2001). At the community level, competitive interactions can lead to competitive exclusion, may alter community structure among co-occurring species (Lamb *et al.* 2009) and can influence plant invasions (Levine 2001; Gurevitch *et al.* 2011, Bennett *et al.* 2014). Thus, competition has the potential to alter fundamental aspects influencing the evolution, persistence and coexistence of species in natural and managed landscapes. Despite the importance of competition at many organizational scales, and despite it being an inherently social interaction, only recently have ecologists explicitly focused on understanding plant competition through a behavioural lens (e.g. Gersani *et al.* 2001; Cahill and McNickle 2011; McNickle and Brown 2014). Here, we build upon behavioural concepts and approaches to better understand how plants alter root growth in the context of social interactions.

In many systems, particularly herbaceous communities such as grasslands, the majority of plant biomass is belowground (Schenk and Jackson 2002). Additionally, when measured, root competition is often a more severe limitation to plant growth than is competition aboveground (Casper and Jackson 1997). Nonetheless, our understanding of plant responses to neighbouring shoots is substantially more advanced (e.g. Smith and Whitelam 1997) than our understanding of plant responses to neighbouring roots (Cahill and McNickle 2011). Better information of how plants alter growth patterns and modify patterns of soil occupancy in response to neighbouring roots should advance our understanding of the causes and consequences of competition and coexistence. By using concepts drawn from the field of behaviour, what a plant does in response to some change in the biotic or abiotic environment (Silvertown and Gordon 1989), one can draw upon a rich conceptual foundation to understand deterministic and plastic growth patterns in plants.

There is substantial evidence that many species of plants have the capacity to alter patterns of root placement in response to neighbours (Schenk 2006; reviewed in Cahill and McNickle 2011). The general patterns found include spatial segregation of neighbouring root systems (Baldwin and Tinker 1972; Brisson and Reynolds 1994; Caldwell *et al*. 1996; reviewed in Schenk *et al*. 1999; Holzapfel and Alpert 2003), over-proliferation of roots in the area of potential interaction (Gersani *et al*. 2001; Maina *et al*. 2002; Padilla *et al.* 2013), along with examples of no response (Litav and Harper 1967; Semchenko *et al*. 2007). Behavioural responses to neighbours appear species specific, and can change as a function of neighbour identity (Mahall and Callaway 1991; Falik *et al*. 2003; Bartelheimer *et al*. 2006; Fang *et al*. 2013). Despite the strong evidence that plants exhibit complexity and contingency in how they occupy and explore the soil environment (Mommer *et al.* 2012), the research performed to date is predominately a series of individual studies with idiosyncratic methods and measures, species selections and variable results. Lacking has been a broadly comparative approach to understanding how plants respond to the roots of neighbours (McNickle and Brown 2014), analogous to efforts to understand how plant roots respond to the spatial distribution of soil nutrients (Campbell *et al*. 1991).

How a plant modifies its occupation of the soil environment in response to a neighbour has important implications for competition for limiting soil resources. Root segregation could result in habitat differentiation, leading to a lack of a ‘shared’ resource pool, and thus enhancing coexistence (Silvertown 2004). In contrast, plants which tend to aggregate roots at the zone of interaction may exaggerate the spatial overlap of soil depletion zones, leading to enhanced competitive interactions (Gersani *et al*. 2001). Though there is no existing theory describing which kinds of species are more or less likely to be segregators, aggregators or non-responders in the context of root interactions, there is a theory available in the context of how plants alter root placement and foraging behaviour in response to patchily distributed soil resources. Campbell *et al*. (1991) predicted that ‘large scale foragers’ (plants with large root systems) will exhibit little ability to precisely place roots in nutrient patches, while smaller scale foragers will have greater ability to finely adjust root distribution. A phylogenetically controlled meta-analysis did not find support for this prediction (Kembel and Cahill 2005). Instead, Kembel and colleagues (2005, 2008) found that foraging precision in relation to nutrients was positively associated with a number of traits typically associated with weediness and ruderal life-history strategies. How size, competitiveness and other plant traits are associated with plant responsiveness to neighbours is unknown.

In this study we experimentally test three specific questions. (i) Are there general patterns in an individual's root behaviour to neighbouring plants among 20 co-occurring grassland species? (ii) Is a plant's root behaviour contingent upon neighbour identity? (iii) What other plants traits are associated with root behavioural strategies? To answer these questions, we visualized roots using a window box apparatus, allowing for root identification and quantification.

## Methods

### Species selection

#### Focal plant species selection

We recognize that there is no single optimum combination of species to be included within a comparative study. As we were predominantly interested in questions related to co-existence, we chose species which potentially co-occur within the native rough fescue (*Festuca hallii* (Vasey) Piper) grasslands near Edmonton, Alberta, Canada. The rough fescue grasslands have been described elsewhere (Lamb and Cahill 2008), with the majority of the biomass consisting of grasses and the majority of diversity being found among the eudicots. In particular, Asteraceae and Poaceae are highly represented in terms of diversity and abundance (Bennett *et al.* 2014), and thus we emphasized species belonging to these two families here.

In total, we included 20 species belonging to six families: Asteraceae (10 species); *Achillea millefolium* L., *Artemesia frigid* Willd., *Artemesia ludoviciana* Nutt., *Erigeron glabellus* Nutt., *Gaillardia aristata* Pursh, *Heterotheca villosa* (Pursh) Shinners, *Solidago missouriensis* Nutt., *Symphyotrichum ericoides* (L.) G.L. Nesom, *Symphyotrichum falcatum* (Lindl.) G.L. Nesom, and *Symphyotrichum laeve* (L.) Á. Löve & D. Löve; Poaceae (five species); *Bouteloua gracilis* (Kunth) Lag. ex Griffiths, *Bromus inermis* Leyss., *Elymus glaucus* Buckley, *Koeleria macrantha* (Ledeb.) Schult., and *Poa pratensis* L; Rosaceae (two species); *Drymocallis arguta* Pursh, and *Geum triflorum* Pursh; Brassicaceae (one species); *Descurainia sophia* (L.) Webb ex Prantl; Fabaceae (one species); *Astragalus agrestis* Douglas ex G. Don; Polygonaceae (one species); *Rumex crispus* L. These species have all been used in other studies conducted by the Cahill lab (Wang *et al.* 2010), grow under growth room conditions and are found in the native grasslands in the area (Bennett and Cahill 2013). These species are representative of the larger species pool at this field site, and as they were not chosen for specific aspects of their growth or abundance, species identity is a ‘random effect’.

Seed was field-collected from multiple, naturally occurring plants at the University of Alberta Roy Berg Kinsella Research Ranch located near Kinsella, Alberta, Canada (53°05N, 111°33W).

#### Neighbour plant species selection

Given the large number of species used in this study, along with the substantial time required to visualize and enumerate root growth (below), it was not feasible to conduct a fully pairwise set of competition trials including all species combinations. Instead, we chose to use a phytometer-based approach (*sensu* Wang et al. 2010).

We chose two species not found in this field site, *Phleum pratense* L., Poaceae, and *Lactuca sativa* L. cv. Esmeralda M.I.., Asteraceae, to serve as neighbour species to our 20 focal species. Our intent in selecting these species was to obtain a generic measure of focal plant response to neighbours, rather than one for which there was potentially a long and co-evolved history. We also chose to include one eudicot and one monocot to limit, for stronger phylogenetic representation. We recognize that results may differ if other species were chosen.

### Experimental design

One individual of each focal species was grown under three neighbour treatments: *P. pratense* neighbour, *L. sativa* neighbour and no neighbour (alone). Due to limits in the rate of processing window boxes for visualization, and the size of our growth room, we used temporal, rather than spatial, replication. Each trial consisted of a single replicate of each focal species (20) × neighbour (3) combination; 60 window boxes in total. Replicates were grown between April 2012 and January 2013, with a trial lasting 30–40 days. Due to varying germination success, as well as occasionally limited root visibility in the photos, each species–neighbour combinations was replicated 2–7 times, with most combinations replicated at least three times.

### Window box design, soil conditions and planting

To enhance our ability to visualize roots, we used a window box design that forces plants to grow in a nearly two-dimensional plane. We recognize that though this general approach has been used previously (e.g. Mahall and Callaway 1991), it results in highly artificial growth conditions. Nonetheless, we believe that the standardization of growth conditions afforded is critical to initial efforts in undertaking a comparative study of root responsiveness.

**Figure 1.**
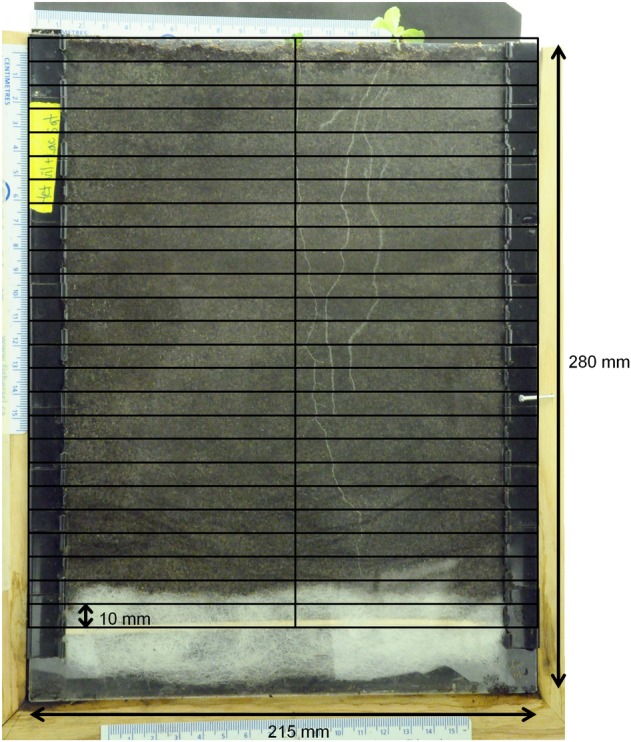
Schematic of experimental window boxes. Soil space available to the plants is ∼5 × 190 × 250 mm. For competition treatments the centre plant is the focal species with the neighbour planted to the right, halfway between the focal plant and box edge. No neighbour plant would be present in the control alone treatment. Overlaid grid shows the depth intervals added for image processing with the centre line delineating the right and left side of focal plant for measures of horizontal asymmetry towards a neighbour (to the right).

Plants were grown in window boxes made of two 215 by 280 mm Plexiglas sheets (one black, one clear) and side spacers (13 mm wide by 5 mm deep) which separated the two Plexiglas sheets creating the soil space (Fig. 1). This configuration was held together with binder clips along the sides. Approximately 30 mm of polyester batting fibre and a horizontal bamboo skewer were arranged at the bottom of each window box to prevent soil leakage yet allowing for drainage. This configuration provided ∼5 × 190 × 250 mm of soil space for plant growth.

Window boxes were filled with a homogeneous soil composition of 3 : 1 sand : topsoil mix, amended with ∼2 % manure by volume. Though we did not perform nutrient analyses on these soils, prior work with similar soils (Wang *et al*. 2010) suggests plant growth would be nutrient limited, particularly nitrogen. Mineral nutrient limitation is also common to the nitrogen-limited soils of the local grasslands from which these seeds were collected (Lamb and Cahill 2008). The top 1 cm of each window box was filled with peat moss (Sun Gro Horticulture Canada Ltd.) to help retain soil moisture.

A single focal species was planted as seed into the centre of each window box, 9.5 cm from each edge. When neighbour plants were used, neighbour seeds were germinated on moist filter paper and bare root transplanted halfway between the focal plant and window box edge. Adding the neighbour plant after germination of the focal plant allowed us to ensure two equally aged seedlings, despite different time-to-germination among species.

### Growth, visualization and harvest

The experiment was conducted under controlled environmental conditions (16 : 8 h light : dark cycle at 24 °C) within a growth room at the University of Alberta Biotron. Window boxes were placed in racks, set to a 40° angle, with the clear side facing down and away from the light source. The angled growing position encouraged more root-Plexiglass contact, enhancing visualization of root growth. To reduce root exposure to light, the clear Plexiglass was covered with a black plastic sheet when roots were not being visualized.

#### Root visualization and harvest

Roots were visualized every 3 days following the germination of the focal plant, for a total of 10 picture sessions. Visualization consisted of photographs taken using a Nikon D80 with shutter priority, shutter speed of 1/30 s and 50 mm focal length. Camera settings, distance and lighting were constant across visualization sessions and replicate trials.

After 27 days of growth, the window boxes were opened, and the neighbour and target plants were removed. Roots and shoots of each plant were separated, rinsed free of soil, dried (48 h at 70 °C) and individually weighed.

#### Image analysis and response variables

All photos were inspected to ensure roots of both individuals (if present) were visible. In the few cases where this was not the case, those replicates were removed from further analysis. Using ArcGIS (v10.1; ESRI) the images were digitized by tracing all roots with lines, and coding each root as belonging to either the focal or neighbour plant. To assist with subsequent analyses, we digitally subdivided each image into twenty-five 10 mm depth intervals. These intervals were further subdivided into ‘left’ and ‘right’ cells, oriented with respect to the main vertical axis of each focal plant (Fig. 1).

We constructed 10 measures for each species indicating its overall behavioural responsiveness to the presence of a neighbour. These included four size-related metrics (aboveground biomass, belowground biomass, total biomass, total root length), two measures of habitat occupancy (total root system area, and maximum root system breadth), three architectural measures (depth of maximum root system breadth, horizontal asymmetry in root length, horizontal asymmetry in root system area) and one measure of relative allocation to root growth (root : shoot biomass ratio). Details of each measure are provided in Table 1. Though other metrics could be calculated, we believe that this suite of measures broadly describes root system architectural responses to neighbours.

**Table 1.** Description of the 10 response measures describing aspects of plant root systems.

Behaviour	Description
Aboveground biomass	Dry mass (g) of all aboveground tissues (g)
Belowground biomass	Dry mass (g) of all belowground tissues (g)
Total biomass	Combined dry mass (g) of aboveground and belowground plant tissues (g)
Root : shoot ratio	Ratio of belowground biomass to aboveground biomass for a given individual
Total root length	Total length of roots (mm) traced using ArcGIS software and attributed to a given individual plant
Total root system area	A convex hull is created around all of the roots of each individual plant. The area of this convex hull (mm^2^) is considered to be the total root system area ‘occupied’ by the plant
Maximum root system breadth	The vertical soil space was divided into 10 mm intervals. For each depth interval the distance (mm) between the farthest root points left and right of centre is calculated. The largest of these widths represents maximum width of the root system
Horizontal asymmetry (root length)	Proportion of total root length for a given individual plant that is found to the right of plant centre. When a neighbour is present, this measure corresponds to the proportion of total root length placed towards that neighbour
Horizontal asymmetry (root system area)	Proportion of total root occupation area for a given individual plant that is found to the right of plant centre. When a neighbour is present, this measure corresponds to the proportion of total occupation area towards that neighbour
Depth of maximum root system breadth	The 10 mm depth interval in which the maximum width is found. The depth measure is the lower end of the interval. For example, a depth of 10 mm would indicate the interval between 0 and 10 mm

### Statistical analysis

#### Multi-species tests

Linear mixed models were used to analyse general patterns in the effects of neighbours on the focal plants, for each of the 10 response variables. Models incorporated planting treatment as a fixed factor (plants grown alone, with *Lactuca sativa* neighbour, or with *Phleum pratense* neighbour) and focal species as a random factor. To meet the assumptions of normality, proportion variables were arcsine transformed; all other variables were ln transformed. Analyses were performed using IBM SPSS Statistics (version 20). Models were also run excluding the random factor, allowing the determination of whether accounting for the variation associated with focal species identity altered model fit based on Hurvich and Tsai's criterion (AICc), accounting for small sample sizes.

*A priori* contrasts [IBM SPSS Statistics (version 20) TEST subcommand in MIXED] were used to determine whether neighbour presence, independent of the identity of the neighbour, altered focal plant response. Only 16 of the 20 focal species had multiple replicates for all three neighbour treatments, and these were included in the analysis.

#### Species-specific responses

To determine how individual species responded to neighbours, we calculated log-response ratios (*sensu* Cahill 1999; Hedges *et al.* 1999) for each of the response variables for each replicate of focal × neighbour combination:

$$\text{LRR}=\text{ln}\left(\frac{V_{N}}{V_{A}}\right)$$

where *V*_N_ is the response value for the focal plant when a neighbour (either *Lactuca sativa* or *Phleum pratense*) was present and *V*_A_ is the response value when the focal plant was grown alone. To calculate LRR, each focal species replicate with a neighbour was paired to an alone plant of the same species based on trial number and resting angle of the boxes. Individual replicates of alone plants were not paired more than once within a neighbour treatment. Positive LRR values indicate an increased response with the neighbour (e.g. increased target plant root biomass); negative values indicate a reduced response with the neighbour (e.g. reduced target plant biomass). One-sample *t*-tests were used to test whether each mean species response ratio was significantly different from zero (no difference between responses with and without neighbour species). Analyses were performed using IBM SPSS Statistics (version 20).

#### Multivariate response and trait correlations

We used principal components analysis (PCA) to explore whether there were multivariate correlations in root responses among species, analogous to larger trait-based studies exploring overall plant strategies (e.g. Grime *et al.* 1997). The PCA was performed using a correlation matrix and equamax rotation in IBM SPSS (version 20). Each species consisted of a single row of data, with its mean LRR for each of the six response variables (LRR) serving as the columns: total biomass, total root length, maximum root system breadth, root : shoot ratio, horizontal asymmetry in root length and depth of maximum root system breadth. We used 6, rather than 10, variables to reduce potential redundancies within the data set.

To test whether a species’ root system size was correlated with its root system responsiveness to neighbours, and if this responsiveness was associated with the degree of competition experienced, we performed four regressions. Root system responsiveness was explored in both the horizontal and vertical dimensions by using the absolute value of each species' mean LRR horizontal asymmetry in root length and LRR depth of maximum root system width, respectively. The absolute value of each species' mean LRR was used in order to analyse the magnitude of root system responsiveness independent of direction. For correlations between root system responsiveness and root system size, the mean of ln belowground biomass of each species when grown alone was used as the dependent variable of size. To test whether a species’ root responsiveness to neighbours was associated with the degree of competitive suppression it experienced, the mean LRR total biomass across both neighbour treatments was used as the independent variable. Regression analysis was performed using IBM SPSS Statistics (version 20).

## Results

### Root response to neighbours

#### General patterns

**Figure 2.**
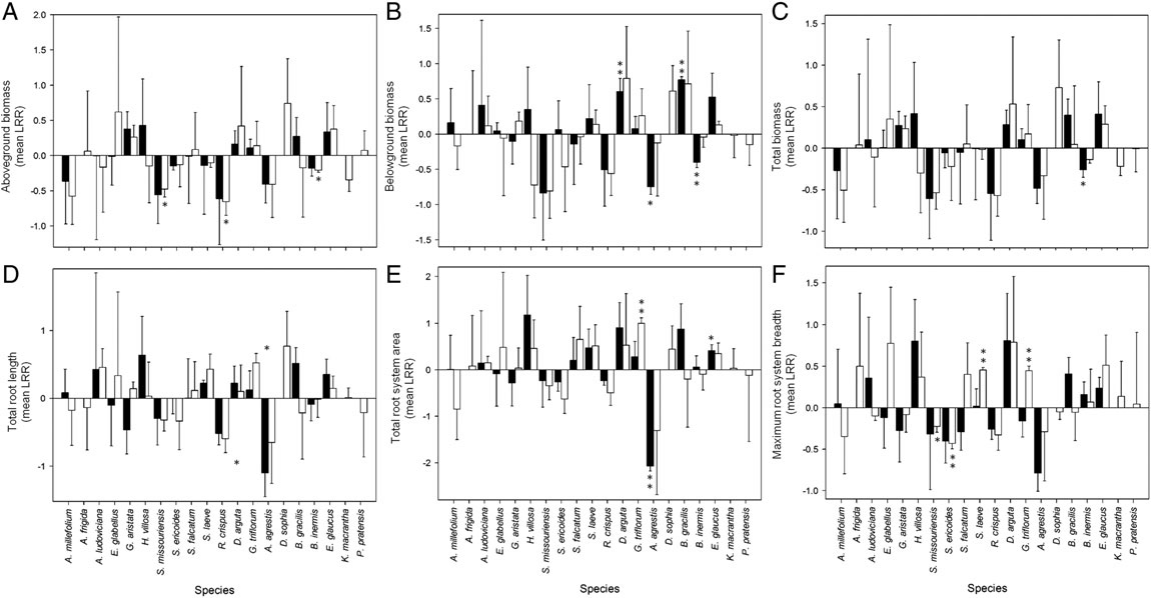
Mean size and habitat occupancy responses (+1 S.E.) of 20 species to neighbour treatment. Graphs show LRR response measures: (A) aboveground biomass, (B) belowground biomass, (C) total biomass, (D) total root length, (E) total root system area, and (F) maximum root system breadth. Closed bars represent the species mean LRR with *Lactuca sativa* neighbour treatment and open bars represent the species mean with *Phleum pratense* neighbour treatment. Asterisks indicate the results of one-sample *t*-tests for a difference from zero (no difference between responses with and without neighbour). *P < 0.10 and **P < 0.05.

**Figure 3.**
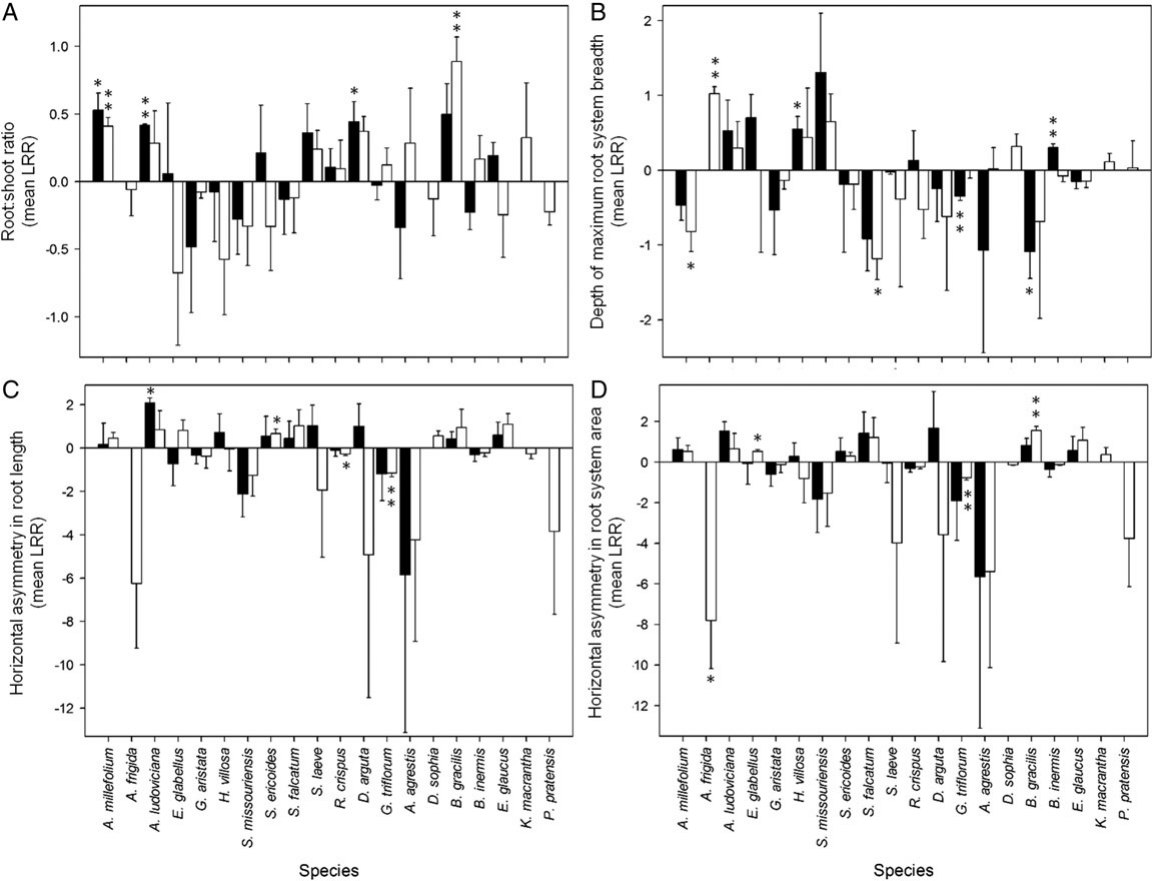
Mean architectural and relative growth allocation responses (+1 S.E.) of 20 species to neighbour treatment. Graphs show LRR response measures: (A) root : shoot ratio, (B) depth of maximum root system breadth, (C) horizontal asymmetry in root length towards neighbour, and (D) horizontal asymmetry in root system area towards neighbour. Closed bars represent the species mean LRR with *Lactuca sativa* neighbour treatment and open bars represent the species mean with *Phleum pratense* neighbour treatment. Asterisks indicate results of one-sample *t*-tests for a difference from zero (no difference between responses with and without neighbour). *P < 0.10 and **P < 0.05.

Across all focal species, there was no overall and consistent effect of the presence of a neighbour on any of the 10 response variables **[see Supporting Information—Table S1]**. However, underneath the lack of a central tendency towards a neighbour effect lies a substantial interspecific variation among the focal species. Including focal plant identity as a random factor in the general linear mixed models substantially increased model fit for 8 of the 10 response variables **[see Supporting Information—Table S1]**, explaining between 20 and 80 % of the variation in a given response variable **[see Supporting Information—Table S1]**. Thus, though on average plants exhibited no root behavioural responses to neighbours, substantial variation in responses occurred among the 20 focal species (Figs 2 and 3). We note that there was no indication, in observation of both roots and shoots, that plants were ‘pot-bound’, nor that space itself was a limiting resource.

#### Species-specific responses

An interspecific variation in root responsiveness to neighbours is seen by examination of the response ratios of each response variable (Figs 2 and 3) **[see Supporting Information—Tables S2–S11]**. For all response variables, neighbours caused increases, decreases or no change, depending upon focal species identity (Figs 2 and 3). Visual examination of Figs 2 and 3 indicate no clear trends in responses across plant groups (eudicot versus monocot) or within families, nor consistent effects of neighbour identity on root system responses. However, 20 species is insufficient to conduct formal phylogenetic analyses, precluding estimates of evolutionary conservatism in root behaviour (*sensu* Kembel and Cahill 2005).

#### Multivariate response

**Figure 4.**
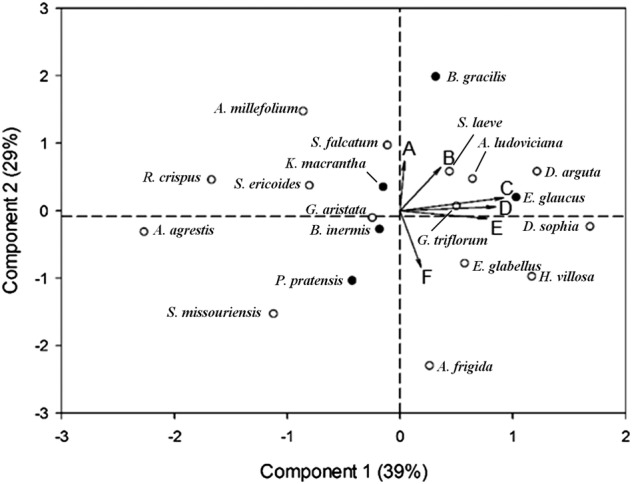
Principal components analysis of six mean response variables (LRR) of 20 species to neighbour treatment. Response variables are: (A) root : shoot ratio, (B) horizontal asymmetry in root length towards neighbour, (C) root length, (D) total biomass, (E) maximum root system breadth, and (F) depth of maximum root system breadth. Component 1 explains 39 % of the variance and Component 2 explains 29 % of the variance.

The six response variables used to describe plant responses to neighbours (total biomass, root : shoot ratio, total root length, horizontal asymmetry in root length, maximum root system breadth and depth of maximum breadth) were reduced to two main axes using PCA, explaining 68 % of the variations in the data (Fig. 4). The first axis (39 %) indicates positive correlations among how a plant's total root length, total biomass and maximum root system breadth respond to the presence of a neighbour. Axis two explains an additional 29 % of the variation in the data, and indicates the responsiveness of a plant's root : shoot biomass ratio and horizontal asymmetry in response to a neighbour are positively correlated with each other, but negatively correlated with a plant's vertical plasticity in response to a neighbour. As before, there was no indication of a consistent difference among monocot and eudicot plant species in how they respond to neighbours.

#### Trait correlations

There was no significant relationship between a species' root system size (ln belowground biomass of species when grown alone) and its root system responsiveness to neighbours in the horizontal (*R*^2^ = 0.074, *F*_1,18_ = 1.436, *P* = 0.246) nor vertical dimensions (*R*^2^ = 0.150, *F*_1,18_ = 3.166, *P* = 0.092). Root system responsiveness to neighbours in the horizontal and vertical dimensions was quantified as mean species LRR horizontal asymmetry in root length and LLR depth of maximum root system width, respectively.

Similarly, neither horizontal (*R*^2^ = 0.017, *F*_1,18_ = 0.303, *P* = 0.589) nor vertical (*R*^2^ = 0.017, *F*_1,18_ = 0.309, *P* = 0.585) root responsiveness were associated with the degree of competitive suppression the focal plant experienced (LRR total biomass).

## Discussion

### General patterns

In previous work, root behavioural responses to neighbours have varied from no response (e.g. Litav and Harper 1967; Semchenko *et al.* 2007), to segregation (e.g. Baldwin and Tinker 1972; Brisson and Reynolds 1994; Caldwell *et al*. 1996; Schenk *et al*. 1999) or over-proliferation (e.g. Gersani *et al*. 2001; Maina *et al*. 2002; Padilla *et al.* 2013). Results presented here (Fig. 1) are consistent with the lack of consistency in these prior findings. We suggest that such behavioural variation is now well demonstrated, and we argue against a strict interpretation of the ‘Tragedy of the Commons’ prediction of over-proliferation of roots in the zone of competitive encounters (Gersani *et al*. 2001). Instead, the variation in behaviour observed here, and in prior studies, is consistent with a broader view that multiple adaptive strategies may occur when plants play competitive games (McNickle and Dybzinski 2013; McNickle and Brown 2014). We also note that neither observing behavioural variation in root responses to neighbours, nor modelling fitness differentials associated with different behavioural types is equivalent to demonstrating these behaviours are adaptive. Again, the study of plant foraging behaviour is substantially behind the understanding of the adaptive value of competitive behaviours aboveground, such as the shade-avoidance response (Dudley and Schmitt 1996). We suggest that more focus on testing the fitness consequences of alternative foraging behaviours is a potentially fruitful area for future research.

Though there was a substantial variation in how plants responded to neighbours, we found no evidence that responses were functionally different among monocots and eudicots. This was surprising, as Kembel and Cahill (2005) found broad differences in the root foraging plasticity of monocot and eudicot species in response to nutrient heterogeneity. Furthermore, both Cahill *et al.* (2008) and Kiær *et al.* (2013) showed different competitive effects among monocots and eudicots, and thus we had expected to see clustering of these two groups in terms of behaviour in response to neighbours. We are unable to determine whether our lack of response was due to our relatively limited phylogenetic representation (only 20 species), or whether our results indicate a lack of phylogenetic bias in the tendency to alter root behaviour in response to neighbours.

Similarly, we also found no consistent effect of neighbour identity on root responsiveness to a neighbour. Although previous studies have not always included neighbour identity as a variable for investigation, when they have the comparison is usually between inter- and intra-specific competition (Mahall and Callaway 1991; Bartelheimer *et al.* 2006) or genotypes of the same species (Callaway and Mahall 2007; Dudley and File 2007; Murphy and Dudley 2009; Fang *et al.* 2013). Evidence suggests that some plants are able to identify their neighbours at the root level (Chen *et al.* 2012), and that some species can alter their root responses according to that identity (Mahall and Callaway 1991; Bartelheimer *et al.* 2006; Callaway and Mahall 2007; Dudley and File 2007; Murphy and Dudley 2009; Fang *et al.* 2013). It is unclear why we found no similar effect here; though caution that it is difficult to draw strong conclusions, as only two neighbour species were used.

### Species-level responses and root behavioural strategies

As mentioned previously, we chose these 20 focal species to be representative of the species that co-occur in a local grassland; they were not chosen to test species-specific hypotheses regarding behavioural responses and strategies. Consequently, each species received relatively little replication, with the strength of the data coming from the comparisons among species. Though these data can be used to test a number of ecologically relevant questions [e.g. are specific root behavioural types associated with high/low abundance in natural system; do specific behavioural types influence a species' response to other ecological challenges (e.g. herbivory)], such questions are well beyond the scope of this manuscript. Instead, we limit our discussion to the two novel behavioural strategies we have identified which are used by plants in response to growing with a neighbour (Fig. 4): size-sensitivity and location-sensitivity.

#### Size-sensitivity

Nearly 40 % of the variations in species' root responses to neighbours were driven by changes in three size-related traits (total root length, change in maximum root system breadth and change in total biomass; Fig. 4). Not surprisingly, these were all positively correlated and indicate an overall reduction in plant size in response to growth with neighbours (i.e. net effects of competition). It is important to recognize, however, that associated with this reduction in plant size is also a reduction in the area of soil occupied by an individual's root system. Depending upon the allometry of these changes within an individual at the community level, there could be important implications for plant neighbourhood size, biomass distributions in the soil, the degree to which pools of limiting resources are shared among neighbours, as well as resource and host availability for mutualists and other members of the soil community. We suggest that this perspective on the ecological importance of shifts in soil occupancy patterns due to social interactions is overlooked within plant ecology, though widely recognized in the context of animal territoriality, density and resource availability (Hixon 1980).

#### Location sensitivity

Not all focal species became smaller in response to growth with neighbours, such that there was no main effect of the presence or absence of neighbours for any response variable, including biomass measures **[see Supporting Information—Table S1]**. However, a lack of biomass effect does not equate to a lack of response to neighbours (Fig. 4). We found nearly 30 % of the variations in root responses to neighbours were associated with changes in biomass allocation (R : S ratio) and fine-scale changes in root placement (horizontal asymmetry and depth of maximum root system breadth), rather overall size. These changes indicate a second root system strategy incorporating behavioural plasticity, rather than simply gross biomass responses. We suggest that this is a potentially critical finding, as it highlights that the impacts of neighbours extend further than the traditionally studied resource limitation-biomass reduction paradigm. These data highlight a potential need to begin more robust exploration of the ‘non-resource’ consequences of neighbours on plant growth and coexistence, analogous to the rapidly increasing research into the non-consumptive effects of predators on prey populations (e.g. Peckarsky *et al.* 2008).

The ability of plants to modify the fine-scale vertical and horizontal placement of roots in response to neighbours is well established (e.g. Mahall and Callaway 1991; Cahill *et al.* 2010; Mommer *et al.* 2010), and has a number of consequences for coexistence, invasion and ecosystem processes. Segregation of the roots of neighbouring plants has long been argued to be a mechanism allowing for species coexistence (Parrish and Bazzaz 1976; Berendse 1983, Craine et al. 2005), due to a reduction in the intensity of competition. The findings here suggest that such a differentiation in micro-scale habitat need not to occur only due to fixed traits of plants (e.g. deep- versus shallow-rooted species), but that behavioural modifications in response to local conditions are not uncommon among plant species. We suggest that reliance on fixed plant traits as a means of understanding the functional ecology of plants can lead to a significant misunderstanding of the mechanisms by which plants can interact with other plants and their environment. We suggest that location-sensitivity behaviours are a potential mechanism that could lead to enhanced coexistence and altered ecosystem functions, even in the face of a strong competitor. It may also be one potential mechanism by which plants are able to tolerate (in a fitness context), growing with aggressive neighbours.

We found no support for the idea that our measures of root responsiveness were related to either plant size (*sensu* the scale and precision ideas of Campbell *et al.* 1991), nor were they associated with the competition experienced by the focal plants. However, we believe that more work focussed on these root responsive strategies is needed, particularly in the context of fitness consequences, competitive tolerance and avoidance, community assembly and ecosystem function. We also agree with McNickle and Brown (2014) who suggest the accumulation of more and of different types of root trait data allows for novel insights into how plants forage and interact in the soil environment.

We note several limitations in our identification of root responsiveness strategies, including a relatively small number of species (though more than have been used before), nearly two-dimensional growing conditions, short duration of the experiment, use of seedlings rather than mature plants and limited replication within species. How these strategies relate to fitness, the ability to perform in the presence of other ecological processes and non-foraging plant traits is also not known.

## Conclusions

Here we used a comparative approach to identify novel behavioural strategies in how plants alter root growth in response to neighbours. Our findings highlight the need to consider species identity when predicting response to neighbours, rather than expect a single dominant strategy of over-proliferation, avoidance or neutrality. Instead, all of these behavioural responses were observed among different species. Though such idiosyncratic responses increase the difficulty of understanding, they do indicate it is critical to understand the biology of the specific species involved in any social interaction. We confirmed prior findings that some species have the potential to alter their fine-scale horizontal and vertical root placement behaviour in response to neighbours, even without showing a negative growth consequence of the ‘competitor’. This potentially has important implications for species coexistence, and may be a behavioural trait-filter influencing community assembly and ecosystem function.

## Sources of Funding

This work was supported by a Discovery Grant, and Discovery Accelerator, awarded by the Natural Sciences and Engineering Research Council of Canada to J.F.C.

## Contributions by the Authors

Both authors conceived of the research and made substantial contributions to the manuscript. P.R.B. conducted the experimentation and analyses.

## Conflict of Interest Statement

None declared.

## Supporting Information

The following additional information is available in the online version of this article –

**Table S1.** Results of general linear mixed model analysis of the fixed factor neighbour treatment (alone, *Lactuca sativa*, or *Phleum pratense*) on 10 response variables with focal species included as a random factor. A change in the AICc value is obtained when focal species is included as a random factor in the analysis. *A priori* contrast of response to neighbours tests alone (1) versus either *Lactuca sativa* (−0.5) or *Phleum pratense* (−0.5) neighbours.

**Table S2.** One-sample *t*-tests for the difference between the mean log-response ratio for aboveground biomass (when grown with neighbour) and zero (indicating no response to neighbour).

**Table S3.** One-sample *t*-tests for the difference between the mean log-response ratio for belowground biomass (when grown with neighbour) and zero (indicating no response to neighbour).

**Table S4.** One-sample *t*-tests for the difference between the mean log-response ratio for total biomass (when grown with neighbour) and zero (indicating no response to neighbour).

**Table S5.** One-sample *t*-tests for the difference between the mean log-response ratio for total root length (when grown with neighbour) and zero (indicating no response to neighbour).

**Table S6.** One-sample *t*-tests for the difference between the mean log-response ratio for root system area (when grown with neighbour) and zero (indicating no response to neighbour).

**Table S7.** One-sample *t*-tests for the difference between the mean log-response ratio for maximum root system breadth (when grown with neighbour) and zero (indicating no response to neighbour).

**Table S8.** One-sample *t*-tests for the difference between the mean log-response ratio for root : shoot ratio (when grown with neighbour) and zero (indicating no response to neighbour).

**Table S9.** One-sample *t*-tests for the difference between the mean log-response ratio for horizontal asymmetry in root length towards neighbour (when grown with neighbour) and zero (indicating no response to neighbour).

**Table S10.** One-sample *t*-tests for the difference between the mean log-response ratio for horizontal asymmetry in root system area towards neighbour (when grown with neighbour) and zero (indicating no response to neighbour).

**Table S11.** One-sample *t*-tests for the difference between the mean log-response ratio for depth of maximum root system breadth (when grown with neighbour) and zero (indicating no response to neighbour).

Additional Information
