# Supplementary material for: Disentangling root system responses to neighbours: identification of novel root behavioural strategies
Source: AoB Plants. 2015 May 27;7:plv059. doi: 10.1093/aobpla/plv059 (PMC4512042; doi:10.1093/aobpla/plv059)
Supplement: Additional Information [file supp_7_plv059_index.html]

Disentangling root system responses to neighbours: identification of novel root behavioural strategies — Additional Information 

# Disentangling root system responses to neighbours: identification of novel root behavioural strategies

## Additional Information

Additional Information

- Supplementary Table 1 - docx file
- Supplementary Table 2 - docx file
- Supplementary Table 3 - docx file
- Supplementary Table 4 - docx file
- Supplementary Table 5 - docx file
- Supplementary Table 6 - docx file
- Supplementary Table 7 - docx file
- Supplementary Table 8 - docx file
- Supplementary Table 9 - docx file
- Supplementary Table 10 - docx file
- Supplementary Table 11 - docx file
