# Supplementary material for: Disentangling root system responses to neighbours: identification of novel root behavioural strategies
Source: AoB Plants. 2015 May 27;7:plv059. doi: 10.1093/aobpla/plv059 (PMC4512042; doi:10.1093/aobpla/plv059)
Supplement: Additional Information [file supp_plv059_plv059supp_table1.docx]

Table S1. Results of general linear mixed model analysis of the fixed factor neighbour treatment (alone, *Lactuca sativa*, or *Phleum pratense*) on 10 response variables with focal species included as a random factor. Change in AICc value is obtained when focal species is included as a random factor in the analysis. *A priori* contrast of response to neighbours tests alone (1) versus either *Lactuca sativa* (-0.5) or *Phleum pratense* (-0.5) neighbours.

|  | Neighbour treatment  (fixed factor) | | |  | Focal species included (random factor) | |  | *A priori* contrast of alone versus  *L. sativa* & *P. pratense* neighbours | | | |
| --- | --- | --- | --- | --- | --- | --- | --- | --- | --- | --- | --- |
| Response variable | df | *F* | *p* |  | Change in AICc | % of variance explained by focal species |  | Estimate | df | *t* | *p* |
| Aboveground biomass | 2, 136.112 | 0.472 | 0.625 |  | -129.172 | 71.84 |  | 0.046169 | 136.169 | 0.486 | 0.627 |
| Belowground biomass | 2, 136.078 | 0.571 | 0.567 |  | -174.233 | 80.06 |  | -0.022935 | 136.115 | -0.225 | 0.822 |
| Total biomass | 2, 136.084 | 0.604 | 0.548 |  | -160.145 | 77.88 |  | 0.024208 | 136.126 | 0.266 | 0.791 |
| Total root length | 2, 136.209 | 0.052 | 0.949 |  | -100.500 | 63.84 |  | -0.008342 | 136.290 | -0.095 | 0.924 |
| Total root system area | 2, 136.227 | 0.153 | 0.858 |  | -90.532 | 60.86 |  | -0.048216 | 136.318 | -0.367 | 0.715 |
| Maximum root system breath | 2, 136.241 | 0.752 | 0.473 |  | -77.003 | 56.63 |  | -0.069496 | 136.349 | -0.798 | 0.426 |
| Root:shoot ratio | 2, 136.392 | 0.519 | 0.596 |  | -63.170 | 50.34 |  | -0.066268 | 136.527 | -0.945 | 0.346 |
| Horizontal asymmetry in root length | 2, 151.000 | 0.327 | 0.721 |  | 2.054 | 0.00 |  | -0.021461 | 151.000 | -0.436 | 0.664 |
| Horizontal asymmetry in root system area | 2, 140.322 | 0.381 | 0.684 |  | 2.054 | 0.06 |  | -0.023802 | 141.616 | -0.471 | 0.638 |
| Depth of maximum root system breadth | 2, 137.442 | 1.864 | 0.159 |  | -11.796 | 19.64 |  | 0.173350 | 137.860 | 1.586 | 0.115 |

*All response variables were ln transformed except proportion of root length and Proportion of area which were arcsine(√) transformed.
