# Supplementary material for: Disentangling root system responses to neighbours: identification of novel root behavioural strategies
Source: AoB Plants. 2015 May 27;7:plv059. doi: 10.1093/aobpla/plv059 (PMC4512042; doi:10.1093/aobpla/plv059)
Supplement: Additional Information [file supp_plv059_plv059supp_table6.docx]

Table S6. One-sample t-tests for difference between mean log response ratio for root system area (when grown with neighbour) and zero (indicating no response to neighbour). Each species and treatment combination was analyzed separately. Bold values indicate p < 0.10.

|  | LRR Total Root System Area | | | | | | | | | | |
| --- | --- | --- | --- | --- | --- | --- | --- | --- | --- | --- | --- |
|  | *Lactuca sativa* neighbour | | | | |  | *Phleum pratense* neighbour | | | | |
| Focal species | Estimate | S.E. | df | *t* | *p* |  | Estimate | S.E. | df | *t* | *p* |
| *Achillea millefolium* | 0.0037 | 0.73909 | 2 | 0.005 | 0.996 |  | -0.8461 | 0.65433 | 2 | -1.293 | 0.325 |
| *Artemesia frigida* | - | - | - | - | - |  | 0.0819 | 1.08828 | 2 | 0.075 | 0.947 |
| *Artemesia ludoviciana* | 0.1440 | 1.12617 | 1 | 0.128 | 0.919 |  | 0.1505 | 0.13758 | 2 | 1.094 | 0.388 |
| *Erigeron glabellus* | -0.0853 | 0.69860 | 2 | -0.122 | 0.914 |  | 0.4826 | 1.60205 | 1 | 0.301 | 0.814 |
| *Gaillardia aristata* | -0.2787 | 0.50107 | 3 | -0.556 | 0.617 |  | 0.0360 | 0.43501 | 2 | 0.083 | 0.942 |
| *Heterotheca villosa* | 1.1770 | 0.84293 | 2 | 1.396 | 0.297 |  | 0.4579 | 0.61466 | 2 | 0.745 | 0.534 |
| *Solidago missouriensis* | -0.2333 | 0.57076 | 2 | -0.409 | 0.722 |  | -0.3441 | 0.30149 | 2 | -1.141 | 0.372 |
| *Symphyotrichum ericoides* | -0.2593 | 0.20592 | 2 | -1.259 | 0.335 |  | -0.6314 | 0.30817 | 2 | -2.049 | 0.177 |
| *Symphyotrichum falcatum* | 0.2033 | 0.48982 | 2 | 0.415 | 0.718 |  | 0.6523 | 0.70297 | 2 | 0.928 | 0.451 |
| *Symphyotrichum laeve* | 0.4719 | 0.40268 | 1 | 1.172 | 0.450 |  | 0.5158 | 0.45409 | 1 | 1.136 | 0.460 |
| *Rumex crispus* | -0.2369 | 0.09672 | 1 | -2.449 | 0.247 |  | -0.4939 | 0.27159 | 2 | -1.819 | 0.211 |
| *Drymocallis arguta* | 0.9020 | 0.53352 | 3 | 1.691 | 0.189 |  | 0.5284 | 1.09952 | 1 | 0.481 | 0.715 |
| *Geum triflorum* | 0.2787 | 0.33204 | 2 | 0.839 | 0.490 |  | 1.0013 | 0.11740 | 2 | 8.529 | **0.013** |
| *Astragalus agrestis* | -2.0718 | 0.10868 | 1 | -19.064 | **0.033** |  | -1.3082 | 1.37989 | 2 | -0.948 | 0.443 |
| *Descurainia sophia* | - | - | - | - | - |  | 0.4468 | 0.49617 | 1 | 0.900 | 0.533 |
| *Bouteloua gracilis* | 0.8751 | 0.53400 | 2 | 1.639 | 0.243 |  | -0.1999 | 1.03539 | 2 | -0.193 | 0.865 |
| *Bromus inermis* | 0.0567 | 0.24138 | 2 | 0.235 | 0.836 |  | -0.0965 | 0.33856 | 1 | -0.285 | 0.823 |
| *Elymus glaucus* | 0.4137 | 0.12828 | 2 | 3.225 | **0.084** |  | 0.3478 | 0.22844 | 2 | 1.522 | 0.267 |
| *Koeleria macrantha* | - | - | - | - | - |  | 0.0328 | 0.42276 | 2 | 0.078 | 0.945 |
| *Poa pratensis* | - | - | - | - | - |  | -0.1205 | 1.42810 | 2 | -0.084 | 0.940 |
