# Supplementary material for: Disentangling root system responses to neighbours: identification of novel root behavioural strategies
Source: AoB Plants. 2015 May 27;7:plv059. doi: 10.1093/aobpla/plv059 (PMC4512042; doi:10.1093/aobpla/plv059)
Supplement: Additional Information [file supp_plv059_plv059supp_table8.docx]

Table S8. One-sample t-tests for difference between mean log response ratio for root:shoot ratio (when grown with neighbour) and zero (indicating no response to neighbour). Each species and treatment combination was analyzed separately. Bold values indicate p < 0.10.

|  | LRR Root:Shoot Ratio | | | | | | | | | | |
| --- | --- | --- | --- | --- | --- | --- | --- | --- | --- | --- | --- |
|  | *Lactuca sativa* neighbour | | | | |  | *Phleum pratense* neighbour | | | | |
| Focal species | Estimate | S.E. | df | *t* | *p* |  | Estimate | S.E. | df | *t* | *p* |
| *Achillea millefolium* | 0.5285 | 0.1260 | 2 | 4.196 | **0.052** |  | 0.4090 | 0.0660 | 2 | 6.202 | **0.025** |
| *Artemesia frigida* | - | - | - | - | - |  | -0.0601 | 0.1930 | 2 | -0.311 | 0.785 |
| *Artemesia ludoviciana* | 0.4141 | 0.0117 | 1 | 35.257 | **0.018** |  | 0.2834 | 0.2395 | 2 | 1.183 | 0.358 |
| *Erigeron glabellus* | 0.0594 | 0.5220 | 2 | 0.114 | 0.920 |  | -0.6767 | 0.5341 | 1 | -1.267 | 0.425 |
| *Gaillardia aristata* | -0.4853 | 0.4838 | 3 | -1.003 | 0.390 |  | -0.0775 | 0.044/1 | 2 | -1.727 | 0.226 |
| *Heterotheca villosa* | -0.0766 | 0.3680 | 2 | -0.208 | 0.854 |  | -0.5762 | 0.4101 | 2 | -1.405 | 0.295 |
| *Solidago missouriensis* | -0.2770 | 0.2615 | 2 | -1.059 | 0.400 |  | -0.3314 | 0.2898 | 2 | -1.144 | 0.371 |
| *Symphyotrichum ericoides* | 0.2114 | 0.3524 | 2 | 0.600 | 0.610 |  | -0.3333 | 0.3269 | 2 | -1.020 | 0.415 |
| *Symphyotrichum falcatum* | -0.1332 | 0.2602 | 2 | -0.512 | 0.660 |  | -0.1220 | 0.2589 | 2 | -0.471 | 0.684 |
| *Symphyotrichum laeve* | 0.3611 | 0.2140 | 1 | 1.687 | 0.341 |  | 0.2402 | 0.1385 | 1 | 1.734 | 0.333 |
| *Rumex crispus* | 0.1057 | 0.1377 | 1 | 0.768 | 0.583 |  | 0.0948 | 0.2112 | 2 | 0.449 | 0.697 |
| *Drymocallis arguta* | 0.4414 | 0.1492 | 3 | 2.959 | **0.060** |  | 0.3723 | 0.1099 | 1 | 3.389 | 0.183 |
| *Geum triflorum* | -0.0279 | 0.1081 | 2 | -0.258 | 0.820 |  | 0.1218 | 0.1273 | 2 | 0.957 | 0.440 |
| *Astragalus agrestis* | -0.3423 | 0.3758 | 1 | -0.911 | 0.530 |  | 0.2840 | 0.4066 | 2 | 0.699 | 0.557 |
| *Descurainia sophia* | - | - | - | - | - |  | -0.1284 | 0.2739 | 1 | -0.469 | 0.721 |
| *Bouteloua gracilis* | 0.4968 | 0.2261 | 2 | 2.197 | 0.159 |  | 0.8869 | 0.1823 | 2 | 4.865 | **0.040** |
| *Bromus inermis* | -0.2257 | 0.1305 | 2 | -1.730 | 0.226 |  | 0.1649 | 0.1751 | 1 | 0.942 | 0.519 |
| *Elymus glaucus* | 0.1924 | 0.0964 | 2 | 1.997 | 0.184 |  | -0.2460 | 0.3169 | 2 | -0.776 | 0.519 |
| *Koeleria macrantha* | - | - | - | - | - |  | 0.3244 | 0.4040 | 2 | 0.803 | 0.506 |
| *Poa pratensis* | - | - | - | - | - |  | -0.2244 | 0.0971 | 2 | -2.311 | 0.147 |
